# Evaluating Postural Transition Movement Performance in Individuals with Essential Tremor via the Instrumented Timed Up and Go

**DOI:** 10.3390/s24072216

**Published:** 2024-03-29

**Authors:** Patrick G. Monaghan, William M. Murrah, Harrison C. Walker, Kristina A. Neely, Jaimie A. Roper

**Affiliations:** 1 School of Kinesiology, Auburn University, Auburn, AL 36849, USA; hr8036@wayne.edu (P.G.M.); kaneely@auburn.edu (K.A.N.); 2 Department of Educational Foundations, Leadership, and Technology, Auburn University, Auburn, AL 36849, USA; wmm0017@auburn.edu; 3 Department of Neurology, University of Alabama at Birmingham, Birmingham, AL 35249, USA; 4 Department of Neurosurgery, University of Alabama at Birmingham, Birmingham, AL 35249, USA; 5 Department of Biomedical Engineering, University of Alabama at Birmingham, Birmingham, AL 35249, USA

**Keywords:** mobility, essential tremor, gait, wearable sensor, functional assessment

## Abstract

Flexibility in performing various movements like standing, walking, and turning is crucial for navigating dynamic environments in daily life. Individuals with essential tremor often experience movement difficulties that can affect these postural transitions, limiting mobility and independence. Yet, little research has examined the performance of postural transitions in people with essential tremor. Therefore, we assessed postural transition performance using two versions of the timed up and go test: the standard version and a more complex water-carry version. We examined the total duration of the standard and water-carry timed up and go in 15 people with and 15 people without essential tremor. We also compared the time taken for each phase (sit-to-stand phase, straight-line walk phase, stand-to-sit phase) and the turning velocity between groups. Our findings revealed decreased performance across all phases of standard and water-carry timed up and go assessments. Further, both ET and non-ET groups exhibited reduced performance during the water-carry timed up and go compared to the standard timed up and go. Evaluating specific phases of the timed up and go offers valuable insights into functional movement performance in essential tremor, permitting more tailored therapeutic interventions to improve functional performance during activities of daily living.

## 1. Introduction

Essential tremor (ET) stands as one of the most prevalent movement disorders, affecting 2.2% of the United States population alone [1]. Traditionally, ET has been viewed as a predominantly upper extremity tremor disorder. However, in recent years, the viewpoint of ET being considered a monosymptomatic disorder characterized by upper extremity tremor has been supplanted. Recent studies have highlighted marked mobility impairments in individuals with ET [2,3,4,5,6,7,8,9]. These mobility impairments can contribute independently to functional disability, particularly in elderly patients who take medications that act on tremors and other neurological symptoms. Therefore, a detailed and thorough examination of functional movement performance is warranted in patients with and without ET.

Various clinical assessment tools are available to evaluate dynamic gait and balance in populations with ET and other movement disorders [10]. The timed up and go (TUG) test is a well-established clinical assessment of dynamic gait and balance in populations with movement disorders such as ET [11]. A unique aspect of the TUG assessment is that it comprises a series of complex activities, which can provide insight into one’s ability to perform critical postural transition movements. The investigation of the phase-specific performance of the TUG may enhance clinical evaluation. For example, the examination of the total TUG time does not readily distinguish Parkinson’s disease patients with and without cognitive impairment [12]. However, an assessment of component phases of the TUG, such as the duration of the sit-to-stand phase, was able to provide such distinction [12]. Therefore, it is plausible that the examination of component phases of the TUG may provide additional sensitivity in detecting subtle functional alterations in patients with ET.

Prior work has determined that people with ET display prolonged total TUG time compared with healthy controls [9,13,14,15,16]. While the total duration of the TUG is clinically useful, few studies in an ET population have examined and compared performance on each phase of the TUG. Moreover, few studies have compared phase-specific performance during a more complex version of the assessment. One previous study reported that individuals with ET took longer to perform the sit-to-stand and turning phases of the TUG. However, this study did not compare performance to a non-ET population’s [15]. While the total TUG duration is still a useful outcome metric, measuring the performance of phases of the TUG may yield more distinct features of the disease. Altered performance in specific phases of TUG, such as the sit-to-stand or turning phase, may also indicate deficits in lower extremity strength or dynamic stability, contributing to compromised daily function. Therefore, a more detailed comparison and examination of phase-specific TUG performance between a population with and without ET is warranted.

The primary objective of this study is to compare postural transitions via the instrumented TUG between people with and without ET. Specifically, we compare performance during a standard TUG and a more complex water-carry TUG. In addition to total TUG time, we will also contrast specific phases of the TUG (sit-to-stand, straight-line walk, turning, and stand-to-sit) in people with and without ET. Based on the previous literature [12,15,17], we hypothesize that people with ET will display longer total times and decreased performance in the sit-to-stand and turning phases of both the standard and water-carrying TUG. The outcomes of this study will enhance our understanding of functional mobility performance in people with ET. Ultimately, it may permit more patient-centered therapeutic strategies and enhance the performance of activities of daily living in people with ET.

## 2. Materials and Methods

### 2.1. Participants

All participants provided written informed consent prior to study participation, as approved by the Auburn University Institutional Review Board. Fifteen people with ET and fifteen age-matched people without ET participated in this study. Participants were eligible to participate in the study if they were aged between 18 and 87 and reported being free from lower extremity injuries or surgeries in the past 12 months that may have changed their walking pattern or limited their capacity to complete the protocol. Participants with ET were recruited from the local area via advertised flyers and word of mouth. Study referrals from the neurology clinic at the University of Alabama at Birmingham were also part of the recruitment strategy to maximize the potential number of participants. A convenience sample of age-matched older adults from the local community was also recruited. The ET group reported to the study in their optimal therapeutic state. Participant characteristics can be observed in Table 1.

### 2.2. Experimental Setup

**Figure 1:**
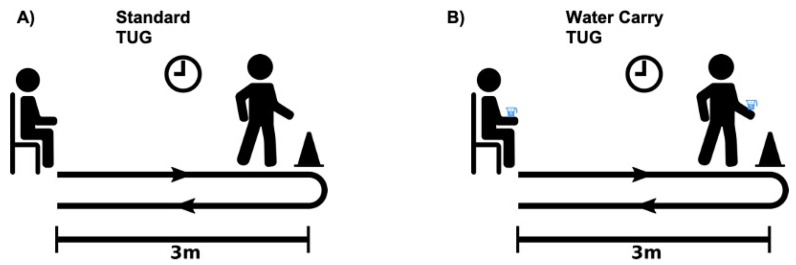
(**A**) Standard and (**B**) water-carry TUG assessments. Participants began seated, rose to a standing position, walked 3 m, performed a 180° turn, walked back 3 m, and then returned to a seated position. For the water-carry TUG assessment, participants performed the same task while carrying a tray with a cup filled with 325 mL of water.

Participants were equipped with six wireless inertial measurement units (Opal, Generation 2, APDM, Inc., Portland, OR, USA), with three-dimensional sensors placed at the sternum and lumbar spine and one at each foot and wrist. Participants then completed a series of instrumented TUG assessments [17,18,19,20]. The instrumented TUG objectively characterizes parameters during postural transitions (sit-to-stand, stand-to-stand, walking, and turning) [18]. Each of these parameters has been previously validated with a motion analysis system in a gait laboratory [17]. Furthermore, the previous literature has also highlighted good test–retest reliability for assessing each of the phases of the TUG [20]. Participants completed two TUG assessments: (1) the standard TUG and a more complex (2) water-carry TUG (Figure 1). Participants completed one trial of each assessment. All assessments were performed using the same armless chair (height 37 cm). The water-carry TUG assessment used a 14 oz paperboard cup filled with 325 mL of water. The same cup was used for all participants.

### 2.3. Standard and Water-Carry TUG

Participants sat with their backs against an armless chair. When the administrator verbally prompted the participant to begin, the participant stood up with their arms across their chest, walked to a piece of tape on the floor three meters away, turned around, and then walked back three meters and returned to a seated position. For the water-carry TUG, participants performed the same task; however, this time, they were instructed to carry a tray with a cup full of water. When prompted, participants once again stood, walked to a piece of tape on the floor three meters away, turned around, and then walked back three meters and returned to a seated position, all while carrying a tray with a cup full of water. For the water-carry TUG assessment, a table was also placed beside the participant to place the tray and the water cup if assistance was needed during the sit-to-stand or stand-to-sit phases of the water-carry TUG assessment.

### 2.4. Data Analysis

**Figure 2:**
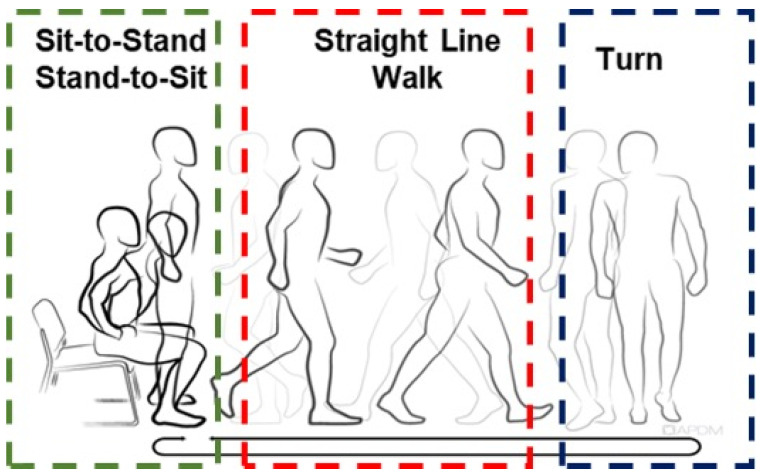
Phases of the timed up and go assessment. The TUG can be divided into four phases: (1) sit-to-stand, (2) straight-line walk, (3) turn, and (4) stand-to-sit. The instrumented TUG uses automated algorithms to characterize durations and outcome measures of the TUG phases objectively.

Data were captured (128 Hz) and processed using the Moveo Explorer data collection software (APDM Inc., Portland, OR, USA). The Moveo Explorer™ software (version 1.0) analyzed the raw data from all sensors using an integrated and automatic algorithm to calculate the durations and outcome measures of the TUG phases. The TUG can be divided into four phases: (1) sit-to-stand, (2) straight-line walk, (3) turn, and (4) stand-to-sit (Figure 2). The sit-to-stand time is the time required to stand up from a seated position at the beginning of the assessment. The straight-line walk time represents the time required to walk 3 m to a piece of the tape on the floor plus the time to return to the chair. Turn velocity indicates the peak angular velocity of the trunk during the turns. As two turns are involved in the TUG assessment, the average turn velocity was used in the analysis. Finally, the stand-to-sit duration is the time required to transition from standing to seated at the end of the trial. Algorithms to detect postural transition movements during the instrumented TUG have been shown to display good sensitivity and test–retest reliability [17,21].

### 2.5. Statistical Analyses

Statistical analyses were performed in IBM SPSS Statistics Version 26 (IBM Corp., Armonk, NY, USA). The distribution of postural transition outcomes from both TUG assessments was assessed using the Shapiro–Wilks test, the inspection of Q–Q plots, and the examination of skewness and kurtosis values. Group comparisons of demographic and clinical characteristics were made using independent sample *t*-tests or chi-square tests of independence. A Group (ET and non-ET) × Condition (standard TUG and water-carry TUG) mixed-effects analysis of variance (ANOVA) with repeated measures examined the effects of Group and Condition and their interaction on postural transition outcomes during specific phases of the standard and water-carry TUG (sit-to-stand, straight-line gait, turning, and stand-to-sit). If significant main effects or interactions were detected in the ANOVA models, post hoc comparisons were corrected using the Bonferroni adjustment for multiple comparisons. If the assumption of sphericity was violated according to Mauchly’s test, Greenhouse–Geisser adjusted degrees of freedom were interpreted. Significance was set at α <0.05 for all analyses.

## 3. Results

A comparison of demographic and clinical characteristics of our ET and non-ET groups can be observed in Table 1.

### 3.1. Phases of Standard and Water-Carry TUG

Mean and standard deviation data for TUG outcomes during phases of a standard and water-carry TUG can be observed in Table 2.

### 3.2. Total TUG Duration

**Figure 3:**
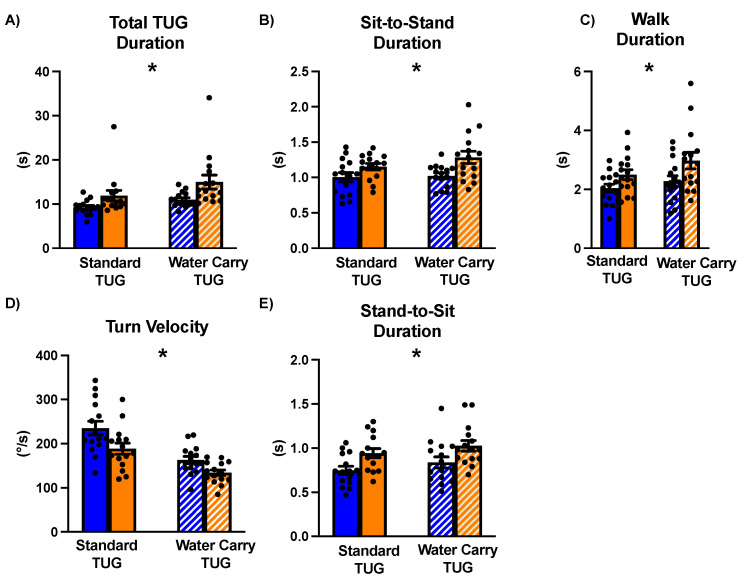
Between-group differences in the standard and water-carry TUG assessments in people with and without essential tremor. (**A**) shows the total duration of each respective TUG, (**B**) displays the sit-to-stand phase, (**C**) represents the straight-line walk phase, (**D**) depicts the turn velocity in the turning phase, and (**E**) represents the stand-to-sit phase. The left side of the graphs indicates the standard TUG assessments, with solid blue bars indicating the non-ET group and solid orange bars indicating the ET group. The right side of the graphs indicates the water-carry TUG assessments, with striped, blue bars indicating the non-ET group and striped, orange bars indicating the ET group. Individual data points are shown as solid black circles. Standard error bars are also depicted. The main effect of the group is denoted by an asterisk (*).

**Figure 4:**
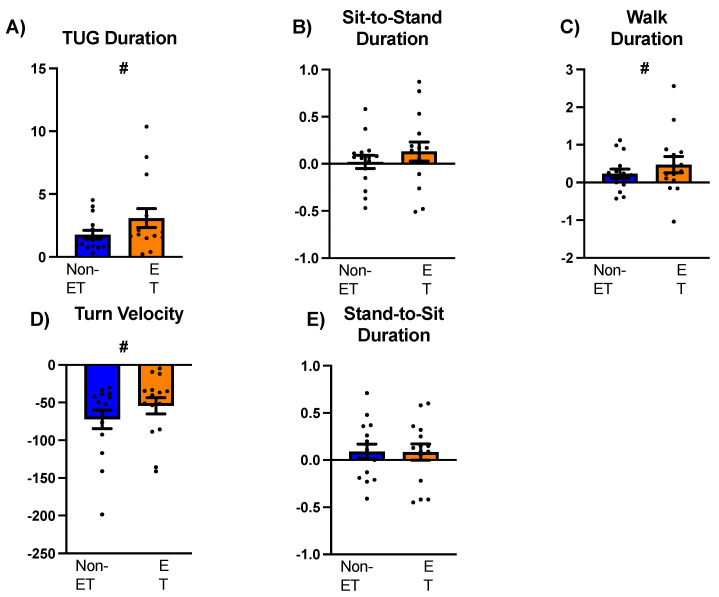
A comparison of performance between standard and water-carry TUGs in individuals with and without essential tremor. Positive values indicate longer/faster performance during the water-carry TUG, while negative values indicate shorter/slower performance during the standard TUG. Panel (**A**): total TUG task duration, Panel (**B**): sit-to-stand phase, Panel (**C**): straight-line walk phase, Panel (**D**): turn velocity in the turning phase, and Panel (**E**): stand-to-sit phase. Blue bars represent the non-ET group, while orange bars represent the ET group. Black-filled circles indicate individual data points. Standard error bars are also depicted. The symbol # denotes the main effect of the condition.

A repeated measures ANOVA compared the total TUG duration between groups (ET and non-ET) across the standard and water-carry conditions. The results revealed a significant main effect of Group (F_1,28_ = 5.62, *p* = 0.03, ŋ^2^ = 0.17). Post hoc comparisons indicated that irrespective of Condition, people with ET took longer to complete the TUG assessments compared to the non-ET group (Mdiff = 3.32, SE = 1.40, *p* = 0.03) (Figure 3A). A significant main effect of Condition was also observed (F_1,28_ = 34.82, *p* < 0.001, ŋ^2^ = 0.55). Irrespective of group, the water-carry TUG assessment took longer to complete compared to the standard TUG assessment (Mdiff = 2.43, SE = 0.41, *p* < 0.001) (Figure 4A). No significant Group X Condition interaction was observed (F_1,28_ = 2.60, *p* = 0.12, ŋ^2^ = 0.09).

### 3.3. Sit-to-Stand Phase

Our analysis investigating the sit-to-stand performance between people with and without ET during the standard and water-carry TUG revealed a main effect of Group (F_1,27_ = 10.1, *p* = 0.004, ŋ^2^ = 0.27). Specifically, people with ET took longer to complete this phase of the TUG compared to their non-ET counterparts (Mdiff = 0.209, SE = 0.07, *p* = 0.004) (Figure 3B). No significant main effect of Condition was observed (F_1,27_ =1.185, *p* = 0.29, ŋ^2^ = 0.04) (Figure 4B) nor was there a significant main Group x Condition interaction (F_1,27_ = 0.597, *p* = 0.45, ŋ^2^ = 0.02).

### 3.4. Straight-Line Walk Phase

A main effect of Group was observed (F_1,28_ = 5.05, *p* = 0.03, ŋ^2^ = 0.15). People with ET took longer to complete the walk phases compared to their non-ET counterparts irrespective of Condition (Mdiff = 0.574, SE = 0.23, *p* = 0.04) (Figure 3C). We found a significant main effect of Condition (F_1,28_ = 8.332, *p* = 0.007, ŋ^2^ = 0.23). Regardless of Group, participants walked slower during the water-carry TUG compared to the regular TUG (Mdiff = 0.355, SE = 0.12, *p* = 0.007) (Figure 4C). No significant Group X Condition interaction was observed (F_1,28_ = 0.968, *p* = 0.33, ŋ^2^ = 0.03).

### 3.5. Turning Phase

Regarding turning velocity, we report a main effect of Group (F_1,28_ = 7.56, *p* = 0.01, ŋ^2^ = 0.21). People with ET walked slower than non-ET counterparts irrespective of Condition (Mdiff = 37.7, SE = 13.7, *p* = 0.01) (Figure 3D). We also found a significant main effect of Condition (F_1,28_ = 60.18, *p* < 0.001, ŋ^2^ = 0.68). Regardless of Group, participants turned slower during the water-carry TUG compared to the regular TUG (Mdiff = 63.2, SE = 8.2, *p* < 0.001) (Figure 4D). No significant Group X Condition interaction was observed (F_1,28_ = 0.1.24, *p* = 0.28, ŋ^2^ = 0.04).

### 3.6. Stand-to-Sit Phase

Regarding stand-to-sit duration, we report a main effect of Group (F_1,27_ = 11.8, *p* = 0.002, ŋ^2^ = 0.30). People with ET walked slower than non-ET counterparts irrespective of Condition (Mdiff = 0.18, SE = 0.05, *p* = 0.002) (Figure 3E). No effect of Condition was observed (F_1,27_ = 2.71, *p* = 0.11, ŋ^2^ = 0.09). No significant Group X Condition interaction was observed (F_1,27_ = 0.02, *p* = 0.90, ŋ^2^ = 0.001) (Figure 4E).

## 4. Discussion

This study compared postural transitional movements in people with and without ET during a standard and progressively more complex measure of mobility. We report two main findings: (1) people with ET showed decreased performance in each phase of the TUG compared to people without ET and (2) task complexity did not differentially impact TUG performance in people with ET. These results support our hypothesis that people with ET would exhibit differences in TUG phases compared to those without ET. This is among the first studies to examine phase-specific differences during a standard and complex TUG assessment in ET. Our study provides additional insight indicating particular functional movement alterations in people with ET.

Our study is novel as we corroborate and expand upon previous studies by comparing the TUG subcomponent phases to patients without ET [9,13,15,22]. People with ET exhibited decreased performance in each phase of the standard and water-carry TUG assessment compared to those without ET. For instance, during the standard TUG assessment, compared to the non-ET group, people with ET took 0.12 s longer to complete the sit-to-stand phase, 0.46 s longer to complete the straight-line walk phase, and 0.45 s longer to complete the stand-to-sit phase, while turning velocity was 45°/s slower. Similarly, regarding the complex TUG assessment, the ET group took 0.27 s, 0.46 s, and 0.19 s longer to complete the sit-to-stand phase, the straight-line walk phase, and the stand-to-sit phase, while turning velocity was 19°/s slower. Our study findings support previous research on TUG performance in ET, which shows decrements in overall TUG duration and phase-specific performance [9,13,15,22]. Our outcomes also align with Mostille et al.’s, who reported differences in the TUG’s sit-to-stand, turning, and stand-to-sit phases [15]. However, this study exclusively examined ET and failed to compare performance to a non-ET population. Therefore, our study is novel and expands upon these findings by comparing the TUG subcomponent phases to participants without ET.

Each phase of the TUG task represents crucial postural transition movements necessary for everyday activities of daily living. Altered performance in specific TUG phases may indicate functional deficits that may compromise daily function. For example, we report decreased performance during the sit-to-stand and turning phase, which may signify movements requiring lower extremity strength, and dynamic balance may be impacted in people with ET [23,24,25]. Critically, decreased performance in these assessments has been associated with disability [26,27] and decreased quality of life [28]. Focusing solely on total TUG duration may limit the interpretative capacity of the assessment, as it fails to provide quantitative outcomes describing components of the assessment that may be most compromised in populations with mobility impairments. Therefore, our study emphasizes the significance of examining individual TUG phases. Examining phase-specific performance may provide more targeted therapeutic approaches to address specific functional performance alterations in people with ET. 

Task complexity, introduced by adding a water-carrying task during the TUG, did not differentially affect overall or phase-specific TUG performance in people with ET. Compared to the standard and regardless of group, the complex TUG resulted in a 2.43 s longer total TUG time, 0.37 s longer straight-line walk phases, and a 63.2°/s slower turning velocity. The sit-to-stand and stand-to-sit phases showed no differences between the standard and complex TUG assessments. Our study aimed to replicate real-life environments and enhance the sensitivity of detecting postural transition deficits in people with ET by incorporating a secondary manual task. This approach builds upon previous research introducing secondary tasks to increase task complexity [29,30,31,32,33]. For example, Lundin and colleagues introduced a water-carrying task during the TUG in frail older adults. They found that a difference of 4.5 s between the standard TUG and the water-carry TUG was sensitive at detecting those frailer and those at fall risk [29]. While previous studies have investigated TUG and phase-specific performance [9,13,15,22], to our knowledge, we are among the first to explore the impact of a secondary manual task on TUG performance in individuals with essential tremor. Despite the typical clinical characteristics of upper extremity tremor and cognitive impairment in ET, both ET and non-ET groups performed similarly during the water-carry TUG assessment. However, our study did not reveal such differences. Therefore, it is plausible that people with ET may have gained assistance from adding the manual task or that it perhaps did not provide a challenging enough stimulus.

There are limitations in our study that should be considered when interpreting the results. First, our measures for the TUG assessment were somewhat limited. We analyzed the timed duration and velocity of the turn for our first aim. However, future studies should explore specific kinematic differences in each phase of the TUG to better explain alterations in performance. Additionally, our assessments only incorporated the 3 m TUG, and we could not detect spatiotemporal metrics of gait during the straight-line walk phases using our inertial sensors. Future studies should consider extending the walkway length to 7 or 10 m to capture additional gait metrics [17]. Furthermore, it is important to acknowledge that medication used by patients with ET might alter their motor performance; however, we consistently ensured that all participants were tested in their therapeutic optimal state. The limited sample size of 30 individuals (15 with ET and 15 without ET) may have affected our outcomes. While this limitation may have influenced our ability to identify interaction effects between group and condition, we believe this limited dataset addresses critical gaps in the existing ET literature. Further, it is important to consider that there are other factors not included within the scope of this study that may have impacted TUG performance (Supplementary Materials), such as depression, anxiety, cognitive impairment, musculoskeletal deconditioning, obesity, and other age-associated comorbidities. Study outcomes provide a necessary first step at enhancing our understanding of functional mobility performance in people with ET. Further, it offers valuable evidence supporting the selection of sensitive clinical measures of functional mobility. Future work should expand current study findings in a larger, more robust sample of people with ET. Despite these limitations, our study outcomes contribute significantly to understanding walking and functional mobility performance in ET.

## 5. Conclusions

People with ET exhibit decreased performance in all phases of the TUG compared to those without ET, indicating functional movement alterations in tasks requiring lower extremity strength and dynamic balance. Our study outcomes provide insight into specific aspects of functional movements that may be most compromised in ET. Ultimately, study outcomes may provide patient-centered therapeutic approaches, increasing one’s ability to complete everyday activities of daily living and improve quality of life.

## Figures and Tables

**Table 1:** Participant characteristics.

	Overall (n = 30)	ET (n = 15)	non-ET Tremor (n = 15)	*p*-Value
Age (years)	66 (15)	66 (16)	66 (15)	0.88
Sex	19 F, 11 M	8 F, 7 M	11 F, 4 M	0.14
Height (m)	1.67 (0.11)	1.71 (0.09)	1.65 (0.1)	0.19
Mass (kg)	74.15 (20.26)	84.64 (24.17)	65.62 (12.76)	**0.04**
TETRAS-ADL	-	17.43 (10.37)	-	-
TETRAS-Motor	-	21.43 (7.00)	-	-
Fall History (F, NF)	9 F, 21 NF	6 F, 9 NF	3 F, 12 NF	**<0.001**
MMSE	28 (1)	28 (2)	29 (1)	0.43
ABC	85.33 (15.61)	80.92(16.61)	89.74 (13.67)	0.12
FES	12.93 (5.41)	14.27(6.57)	11.6 (3.70)	0.19

Note. Values are reported as mean and standard deviation (SD). TETRAS-ADL: The Essential Tremor Rating Assessment Scale-Activities of Daily Living Subscale; kg: kilograms; m: meter; MMSE: Mini-Mental State Exam, TMT: Trails-Making Testing; ABC: Activities-Specific Balance Confidence Scale; FES: Fall Efficacy Scale; F: fall history; NF: no fall history. **Bold** values indicate differences between essential tremor and non-essential tremor groups.

**Table 2:** Mean and standard deviation data for TUG outcomes during standard and water-carry TUGs across ET and non-ET groups.

	non-ET (n = 15)	ET (n = 15)
**Standard TUG**		
Total TUG Duration (s)	9.25 (1.67)	11.91 (4.59)
Sit-to-Stand Duration (s)	1.04 (0.253)	1.16 (0.180)
Straight-Line Walk Duration (s)	2.04 (0.521)	2.50 (0.657)
Turn Velocity (°/s)	235.35 (59.8)	188.48 (49.2)
Stand-to-Sit Duration (s)	0.75 (0.179)	0.924 (0.194)
**Water-Carry TUG**		
Total TUG Duration (seconds)	11.0 (1.70)	15.0 (6.11)
Sit-to-Stand Duration (seconds)	1.02 (0.160)	1.29 (0.336)
Straight-Line Walk Duration (seconds)	2.28 (0.716)	2.97 (1.09)
Turn Velocity (degrees/seconds)	162.97 (32.2)	134.34 (24.5)
Stand-to-Sit Duration (seconds)	0.838 (0.243)	1.03 (0.232)

Note. TUG: timed up and go.

## Data Availability

The data presented in this study are available on request from the corresponding author.

## Supplementary Materials

The following supporting information can be downloaded at: https://www.mdpi.com/article/10.3390/s24072216/s1.
