# Supplementary material for: Evaluating Postural Transition Movement Performance in Individuals with Essential Tremor via the Instrumented Timed Up and Go
Source: Sensors (Basel). 2024 Mar 29;24(7):2216. doi: 10.3390/s24072216 (PMC11014324; doi:10.3390/s24072216)
Supplement: Supplementary file 1 [file sensors-24-02216-s001.zip › sensors-2909975-supplementary.pdf]

Supplemental

Table S1. Correlations between demographic and clinical characteristics and performance from the standard and water carry TUG.
assessments.

Measure	Correlation (95% Confidence Interval)	p-value
**Standard TUG**		
Age	0.437 (-0.114, 0.782)	0.10
TETRAS-ADL	0.357 (-0.231 -0.754)	0.21
TETRAS-Motor	0.224 (-0.341, 0.670)	0.42
ABC	-0.424 (-0.776, 0.130)	0.10
FES	0.494 (-0.04, 0.809)	0.06
MMSE	0.555 (0.04, 0.836)	**0.03**
**Water Carry TUG**		
Age	0.388 (-0.171, 0.758)	.15
TETRAS-ADL	0.612 (0.10, 0.867)	**.02**
TETRAS-Motor	0.583 (0.09, 0.848)	**.02**
ABC	-0.683 (-0.889, -0.247)	**.01**
FES	0.574 (0.07, 0.844)	**.03**
MMSE	0.424 (-0.129, 0.776)	0.11

Note. Spearman rho correlations and 95% confidence intervals for clinical and demographic characteristics of ET sample and total duration of the standard and water-carry TUG assessments. Significant correlations are highlighted in **bold.** TETRAS-ADL: The Essential Tremor Rating Assessment Scale-Activities of Daily Living Subscale; TETRAS-Motor: The Essential Tremor Rating Assessment Scale-Motor Subscale; ABC: Activities Specific Balance Confidence Scale; FES: Fall Efficacy Scale; MMSE: Mini-Mental State Exam.
